# Relationship between dietary patterns and physical performance in the very old population: a cross-sectional study from the Kawasaki Aging and Wellbeing Project

**DOI:** 10.1017/S1368980023000113

**Published:** 2023-06

**Authors:** Tao Yu, Yuko Oguma, Keiko Asakura, Yukiko Abe, Yasumichi Arai

**Affiliations:** 1 Graduate School of Health Management, Keio University, Fujisawa City, Kanagawa, Japan; 2 Sports Medicine Research Center, Keio University, 4-1-1Hiyoshi, Kouhoku-ku, Yokohama City, Kanagawa 223-0061, Japan; 3 Department of Environmental and Occupational Health, School of Medicine, Toho University, Oota-ku, Tokyo, Japan; 4 Center for Supercentenarian Medical Research, Keio University, Shunjuku-ku, Tokyo, Japan; 5 Keio University Faculty of Nursing and Medical Care, Fujisawa City, Kanagawa, Japan

**Keywords:** Ageing population, Dietary patterns, Hand grip strength, Physical performance, Principal components analysis

## Abstract

**Objectives:**

As the world’s population is ageing, improving the physical performance (PP) of the older population is becoming important. Although diets are fundamental to maintaining and improving PP, few studies have addressed the role of these factors in adults aged ≥ 85 years, and none have been conducted in Asia. This study aimed to determine the dietary patterns (DP) and examine their relationship with PP in this population.

**Design:**

This cross-sectional study (Kawasaki Aging and Wellbeing Project) estimated food consumption using a brief-type self-administered diet history questionnaire. The results were adjusted for energy after aggregating into thirty-three groups, excluding possible over- or underestimation. Principal component analysis was used to identify DP, and outcomes included hand grip strength (HGS), timed up-and-go test, and usual walking speed.

**Setting:**

This study was set throughout several hospitals in Kawasaki city.

**Participants:**

In total, 1026 community-dwelling older adults (85–89 years) were enrolled.

**Results:**

Data of 1000 participants (median age: 86·9 years, men: 49·9 %) were included in the analysis. Three major DP (DP1: various foods, DP2: red meats and coffee, DP3: bread and processed meats) were identified. The results of multiple regression analysis showed that the trend of DP2 was negatively associated with HGS (B, 95 % CI –0·35, –0·64, –0·06).

**Conclusions:**

This study suggests a negative association between HGS and DP characterised by red meats and coffee in older adults aged ≥ 85 years in Japan.

With the ageing of the population worldwide, countries are increasing their average life expectancy annually. However, the gap between average and healthy life expectancy is becoming an issue, and physical performance (PP) is considered important because of the need to promote ageing without morbidity and disability^(1,2)^. PP does not mean specific physical functioning but includes a whole range of tests such as hand grip strength (HGS), walking and standing tests, and is a useful predictor of frailty, dependence on activities of daily living, CVD mortality and all-cause mortality^(1–6)^. In addition, PP is certainly an important factor in successful ageing, helping to ensure the quality of life for the older population, which is why the importance of a nutritional focus in its maintenance or even enhancement has been discussed^(1,7–11)^. The association between PP and nutrition (such as protein and vitamin D) have been reported with inconsistent results^(12,13)^. Although the physiological mechanisms may be better studied with single nutrients, the regular diet consists of foods (complexes of several nutrients) in daily life. It is unlikely that single nutrients are consumed as foods, except for supplements. Therefore, a method (such as dietary pattern (DP)) is needed to assess the diet comprehensively^(14,15)^. There are two major methods for identifying DP: a priori DP, which are based on established hypotheses, and posteriori DP, which depend on data^(14)^. An example of the priori DP is the Mediterranean diet score, which has been reported to be associated with PP. It is unclear if it reflects the daily diets in Japan because of its characteristics of the mediterranean diet and regional differences^(9)^. Other studies have assessed adherence to the Japanese Dietary Balance Guide and examined its association with mortality; however, these studies were sensitive to the accuracy of the dietary survey^(16)^. The posteriori DP are techniques that use principal component analysis, factor analysis or cluster analysis to indentify DP. However, the naming of the DP is left to the investigator in each study. In Japan, 285 unique DP have been identified by factor analysis or principal component analysis. After examining their similarity, six major DP were finally identified, including ‘healthy’ DP or ‘western’ DP. Healthy DP is defined differently in different countries but is characterised by a high intake of plant foods (such as legumes and vegetables) and vitamins and minerals at the nutrient level^(17)^. Such as trend towards DP is positively associated with PP tests^(18)^.

With the expected and current increase in the number of adults aged ≥ 85 years, an important point to consider is that while previous studies have examined various DP, most have focused on adults and younger older adults (about 65 years), and very few studies have focused on adults aged ≥ 85 years.

The association between DP and PP in this age group was examined in the UK. The most frequent DP was characterised by red meats, which longitudinally suggested a negative association with HGS^(10)^. Because of age and generational differences in diet, it is difficult to apply evidence from the younger older population to populations aged ≥ 85 years, and due to possible regional differences, there is an immediate need in public health nutrition to identify DP specific to Asian regions (e.g. Japan and China) and their relationship to PP^(19,20)^. Thus, there is little evidence to indicate what DP that age group may have; in other words, little is known about their eating habits at that age. Therefore, this study used data from a population aged ≥ 85 years living in Japan to identify the major DP in the older population in this age group and to examine the association between DP and PP.

## Methods

### Study population

**Fig. 1:**
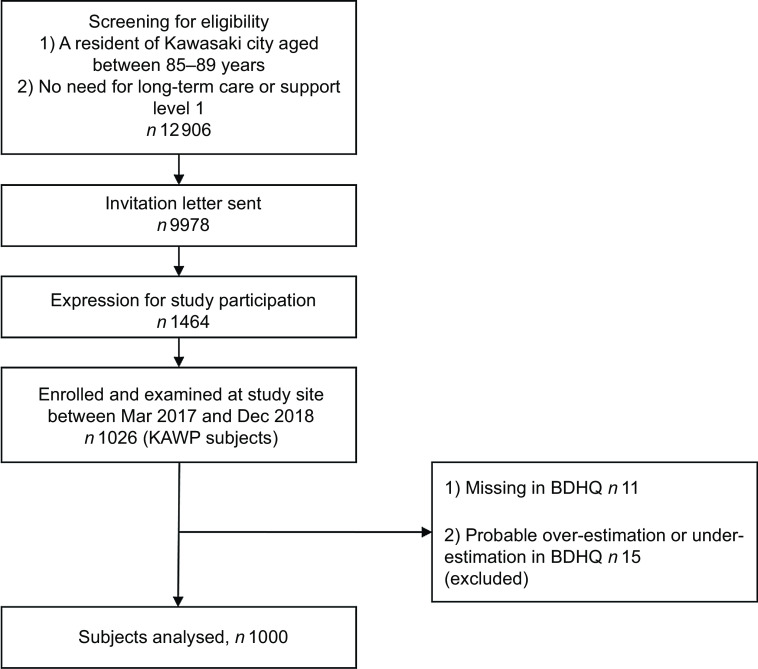
The inclusion criteria of KAWP. There are seven categories of long-term care benefits and support in Japan: no certified (no need for long-term care), support levels 1 and 2 for preventive, long-term care benefits, and care levels 1 to 5 for long-term care benefits. The higher the level of care, the more advanced the functional decline. KAWP, Kawasaki Aging and Wellbeing Project; BDHQ, brief-type self-administered diet history questionnaire

This cross-sectional study used data from the Kawasaki Aging and Wellbeing Project (KAWP) conducted in Kawasaki city (Kanagawa Prefecture, Japan)^(21)^. The inclusion criteria of KAWP were as follows: (1) resident of Kawasaki city (population of 1·5 million), located in the Greater Tokyo Area; (2) age between 85 and 89 years; (3) no need for long-term care or up to support level 1 (no limitations in performing basic activities of daily living); and (4) ability to independently visit the study site (several hospitals in Kawasaki city). Using the basic registration of residents and the long-term care insurance database, 12 906 participants were screened as potential participants. An invitation letter for this study was mailed to 9978 individuals, and 1464 eligible residents expressed their willingness to participate. Between March 2017 and December 2018, 1026 community-dwelling older adults were enrolled in KAWP (Fig. 1). A comprehensive baseline assessment was conducted, which included the assessment of physical, mental, cognitive performance and social participation. The study excluded individuals with deficits in the dietary survey (*n* 11) and those who were deemed to have large reporting errors in the dietary survey (estimated energy intake > 16 736 kJ (4000 kcal) or < 2510·4 kJ (600 kcal), *n* 15).

### Dietary survey

The dietary survey was conducted using the brief-type self-administered diet history questionnaire (BDHQ) that was validated using 3-d half-weighted food records in adults aged ≥ 80 years^(22)^. The BDHQ was completed by the participant but was reviewed and modified as needed by trained researchers. The questionnaire estimates energy and nutrient intake based on the type, amount and frequency of foods consumed in a typical meal during the past month. The BDHQ was sent to the participants’ homes with other questionnaires 2 to 3 weeks before the survey and was completed by them. However, family members were allowed to assist in special cases, such as when the participants could not hold a pen, read or understand. They were brought on the day of the survey, and a trained investigator checked them with the participants on site and made corrections as necessary.

### Test of physical performance

PP assessment included three tests: HGS, timed up-and-go (TUG) test and walking speed – the latter two assessments were performed at the usual speed. HGS was measured twice in the standing position with the dominant hand using a digital dynamometer (Grip D, T.K.K. 5401, Takei Scientific Instruments). The measurement was recorded at a standing position, with the elbow and wrist in the extended and intermediate positions, respectively. Only the dominant hand was measured twice, and the maximum value was used. TUG measures the time from sitting to walking to a marker 3 m in front of the subject and then turning around and sitting down again. Walking speed was averaged as the 5 m walking test time twice at a comfortable speed. We provided 2-m back and forth sections (acceleration and deceleration sections) on the 5-m gait path.

### Assessment of covariates

The study assessed demographic and socio-economic variables such as sex, age, education, economic status and employment status, as well as lifestyle habits such as smoking habits and physical activity. Physical activity was assessed using the modified Zutphen Physical Activity Questionnaire. This questionnaire was validated with the same age population^(23)^. Participants were asked if they had conducted any of the activities (walking and exercise/sports) in the previous week. The number of metabolic equivalents (MET)×hours per week was calculated by multiplying the activity intensity, duration (hours/week) and frequency (number of times per week) of each physical activity. Medical data included medical history (heart disease, kidney disease, hypertension, diabetes, dyslipidaemia and cancer) and long-term care needs. Cognitive performance was assessed by a clinical psychologist in a private room using the Mini-Mental State Examination (MMSE).

### Statistical analysis

Since it is beneficial to identify the characteristics of the population, this study used principal component analysis to identify DP. Before identifying DP, the estimated fifty-eight foods were categorised into thirty-three foods and food groups, which were estimated based on previous studies of 70- to 90-year-old Japanese^(24)^. They were adjusted for energy using the density method, and principal component analysis was performed. Based on the eigenvalues and scree plots, we examined up to the third principal components (DP) for possible interpretation as DP and calculated the principal component score. The principal component score is a continuous variable ranging from –1 to 1. A higher score indicates a higher adherence to the DP. In this study, the median principal component score was used to divide the DP into two groups to indicate the trend towards each DP. The low-trend and high-trend groups were differentiated (low-trend group *v*. high-trend group). A linear regression model was used to examine the relationship between DP and PP. In addition to the principal component scores (quantitative variables) for each DP, Model 1 was adjusted for sex, age, BMI and MMSE. Model 2 was adjusted for activities of daily living, years of education, economic status, smoking habits, physical activity level, living conditions, medical history (CVD, renal disease, hypertension, diabetes, dyslipidaemia and cancer) and long-term care needs in addition to the variables in Model 1. Statistical analysis was performed using SPSS version 26.0 (IBM Japan, Tokyo, Japan), and statistical significance was set at *P* > 0·05.

## Results

Three DP were identified (Tables 1 and 2). The first DP was characterised by the intake of various plant foods. In addition to vegetables (other green and dark yellow vegetables), other plant foods such as mushrooms, seaweed and fruits showed positive loading. Also, foods such as fish and seafood showed positive loadings, and this DP was classified as ‘various foods’ (DP1). The second DP was characterised by consuming protein-rich foods such as meats, eggs and soya products. Of these, red meats had the highest loading. In addition, coffee had a high loading, and this DP was classified as ‘red meats and coffee’ (DP2). The third DP was characterised by a low loading of meshi (cooked rice) and miso soup (traditional Japanese soup) and a high loading of bread and processed meats and was classified as ‘bread and processed meats’ (DP3). Three DP accounted for 10·7, 6·5 and 5·7 % of the variance, respectively (22·9 %). The characteristics of all participants and details of socio-economic variables, lifestyle, and medical information for each DP are shown in Table 2. The total number of participants was 1000 (49·9 % men), and the median age was 86·9 years. For DP1, the high-trend group (i.e. the group with principal component scores higher than the median) had more women and better MMSE scores, and differences in economic status were observed. In terms of lifestyle, differences were found in smoking habits and physical activity in the two groups (high-trend group *v*. low-trend group). DP2 showed significantly fewer people working in the high-trend group; DP3 confirmed more women and longer education in the high-trend group.

**Table 1:** Identification of dietary patterns

	DP1, various foods	DP2, red meats and coffee	DP3, bread and processed meats
Cooked rice	−0·36		−0·63
Noodles			
Bread	−0·20		0·49
Miso soup			−0·51
High-fat milk			
Low-fat milk			
Red meats		0·28	
Chicken		0·20	
Processed meats		0·22	0·36
Fish	0·33		−0·26
Shellfish			
Seafood	0·22		−0·29
Egg		0·23	
Potatoes	0·43		
Soya products	0·38	0·25	−0·26
Green and dark yellow vegetables	0·67		
Other vegetables	0·69	0·22	
Pickled vegetables	0·40		
Salad vegetables	0·53	0·25	0·22
Mushrooms	0·51		
Seaweeds	0·50		
Fruit	0·35		
Confectioneries		−0·60	0·28
Ice cream		−0·34	
Sugar	−0·25	0·62	0·23
Fat and oils	0·29	0·28	0·33
Alcoholic beverages	−0·24		
Green tea			−0·21
Black and oolong tea			0·21
Coffee	−0·28	0·45	0·39
Soft drinks			0·27
Fruit and vegetable juice			0·22
Seasonings		0·66	−0·22
Total	3·52	2·15	1·89
Initial eigenvalues % of the variance	10·66	6·51	5·72
Cumulative %	10·66	17·18	22·90

DP, dietary pattern.

Numbers indicate the loading each food group or food accounts for and items with an absolute value < 0·20 are left blank.

**Table 2:** Participant characteristics

				DP1, various foods					DP2, red meats and coffee					DP3, bread and processed meats				
		All participants (*n* 1000)		Low-trend group (*n* 500)		High-trend group (*n* 500)		*P*-value	Low-trend group (*n* 500)		High-trend group (*n* 500)		*P*-value	Low-trend group (*n* 500)		High-trend group (*n* 500)		*P*-value
		Median	25th–75th percentile	Median	25th–75th percentile	Median	25th–75th percentile		Median	25th–75th percentile	Median	25th–75th percentile		Median	25th–75th percentile	Median	25th–75th percentile	
Sex	Men							< 0·001					0·31					0·02
	*n*	499		296		203			241		258			268		231		
	%	49·9		59·2		40·6			48·2		51·6			53·6		46·2		
Age	years	86·9	85·9–88·2	86·8	85·9–88·2	86·9	85·9–88·2	0·88	86·9	85·9–88·2	86·8	85·9–88·2	0·60	86·8	85·8–88·1	86·9	85·9–88·2	0·30
BMI	BMI, kg/m^2^	23·1	21·1–25·1	23·1	21·1–25·1	23·0	21·1–25·2	0·67	23·1	21·2–25·3	23·1	21·0–25·0	0·59	23·1	21·2–25·2	23·0	21·1–25·1	0·44
MMSE	Mini-Mental State Examination	26	24–28	26	24–28	27	25–29	< 0·01	26	24–28	26	24–28	0·95	26	24–28	26	24–29	0·31
ADL (997)	Activities of daily living, Barthel index	100	100–100	100	100–100	100	100–100	0·13	100	100–100	100	100–100	0·52	100	100–100	100	100–100	0·83
Education	Years	11	9–14	11	8–14	11	9–14	0·39	11	9–14	11	9–13	0·76	10	8–13	12	10–14	< 0·001
		*n*	%	*n*	%	*n*	%		*n*	%	*n*	%		*n*	%	*n*	%	
Living status (983)	Living alone			146	29·7	131	26·7	0·32	135	27·4	142	29·0	0·62	128	26·0	149	30·3	0·14
Economic status (974)																		
Very good/good		590	60·6	275	56·6	315	64·5	0·04	302	62·0	288	59·1	0·44	291	60·0	299	61·1	0·78
Neither		200	20·5	110	22·6	90	18·4		92	18·9	108	22·2		104	21·4	96	19·6	
Bad/very bad		184	18·9	101	20·8	83	17·0		93	19·1	91	18·7		90	18·6	92	19·2	
Smoking habits (997)																		
Smoker		42	4·2	30	6·0	12	2·4	< 0·001	19	3·8	23	4·6	0·76	22	4·4	20	4·0	0·91
Ex-smoker		375	37·6	212	42·5	163	32·7		185	37·1	190	38·1		189	38·0	186	37·3	
Non-smoker		580	58·2	257	51·5	323	64·9		294	59·0	286	57·3		287	57·6	293	58·7	
Physical activity level (995)	METs × h/week							< 0·01					0·98					0·67
	Median	15·9		13·9		16·9			15·7		16·0			15·1		16·3		
	25th–75th percentile	8·8–26·3		7·4–25·7		9·8–26·8			8·8–27·1		8·7–25·3			8·4–26·6		9·0–25·6		
Employment status (988)	Working	141	14·3	73	14·7	68	13·8	0·72	91	18·3	50	10·2	< 0·001	68	13·8	73	14·7	0·72
Disease history (963)	The ratio of who have no disease	143	14·9	67	13·8	76	15·9	0·49	71	14·8	72	14·9	0·48	72	14·9	71	14·8	0·72
Need for long-term care (977)	Yes	113	11·6	60	12·3	53	10·9	0·55	56	11·4	57	11·7	0·92	48	9·8	65	13·4	0·09

DP, dietary pattern.

The effective number of participants is shown next to the item. The values are shown as median (25th–75th percentile) or number (%). The *P*-value is a test of the difference between the high- and low-trend groups for each dietary pattern.

The nutritional intake of each DP is shown in Table 3. For macronutrients in DP1, the high-trend group had higher protein and fat intake and lower carbohydrate intake. DP2 was similar to DP1, as protein and fat intake were significantly higher in the high-trend group. The total protein and animal protein intake were high in the high-trend group, while plant protein was not significantly different between the two groups. In other words, the proportion of animal protein intake was higher in the high-trend group. In DP3, total protein, animal protein and plant protein intake were significantly lower in the high-trend group. The intake of total fat, SFA, MUFA, PUFA were significantly higher in the high-trend group, and there was no significant difference in carbohydrate intake between the two groups. Next, micronutrients with antioxidant properties such as vitamins A, E and C were all higher in the high-trend group in DP1 and DP3. In contrast, vitamin C was not significantly different in the two groups in DP2. Most of the other nutrients showed higher values in the high-trend group for DP1 and DP2, but many nutrients showed less variability in DP2 than in DP1. DP3 showed different variability for different nutrients. The association between each DP and PP is shown in Table 4. The DP1 (‘various foods’) and the DP3 (‘bread and processed meats’) were not significantly associated with any outcomes. The DP2 (‘red meats and coffee’) had a significant negative association with HGS even after adjusting for confounders (B: –0·35, 95 % CI –0·63, –0·06). Participants with possible cognitive decline (MMSE score ≤ 21) were excluded from sensitivity analysis, and the relationship with PP was examined by identifying DP, but the results did not change. In addition, when the outcome was changed to HGS per body weight (kg/kg), the significance remained unchanged.

**Table 3:** Nutrient intake in each dietary pattern

		DP1, various foods					DP2, red meats and coffee					DP3, bread and processed meats				
		Low-trend group (*n* 500)		High-trend group (*n* 500)		*P*-value	Low-trend group (*n* 500)		High-trend group (*n* 500)		*P*-value	Low-trend group (*n* 500)		High-trend group (*n* 500)		*P*-value
Protein	%Energy	15·4	13·7–17·0	18·4	16·3–20·4	< 0·001	15·6	13·9–18·0	18·0	15·9–20·2	< 0·001	17·2	15·0–19·9	16·3	14·5–18·4	< 0·001
Animal protein	%Energy	8·8	7·1–10·6	11·5	9·3–13·7	< 0·001	8·9	7·1–11·0	11·3	9·0–13·5	< 0·001	10·1	8·0–13·0	9·8	7·8–11·9	0·02
Plant protein	%Energy	6·5	5·8–7·2	6·9	6·2–7·6	< 0·001	6·7	5·9–7·3	6·7	6·0–7·4	0·48	6·8	6·0–7·6	6·5	5·9–7·2	< 0·001
Fat	%Energy	27·5	23·5–30·6	31·3	28·4–34·0	< 0·001	28·4	24·9–31·3	30·7	27·3–33·9	< 0·001	27·5	23·8–31·2	31·0	28·2–34·1	< 0·001
SFA	%Energy	7·7	6·4–8·9	8·5	7·5–9·3	< 0·001	8·1	6·9–9·2	8·1	6·8–9·1	0·69	7·5	6·2–8·6	8·6	7·7–9·7	< 0·001
MUFA	%Energy	9·6	8·2–10·9	11·0	9·9–12·1	< 0·001	9·9	8·5–11·1	10·9	9·4–12·1	< 0·001	9·5	8·1–10·8	11·1	9·9–12·3	< 0·001
PUFA	%Energy	6·3	5·5–7·2	7·4	6·6–8·2	< 0·001	6·4	5·5–7·1	7·5	6·5–8·2	< 0·001	6·7	5·7–7·7	7·0	6·1–7·9	< 0·01
Carbohydrate	%Energy	53·3	47·6–58·2	48·7	45·0–53·3	< 0·001	53·4	48·4–57·8	48·2	44·3–53·1	< 0·001	51·3	45·6–56·8	50·6	46·3–55·1	0·23
Dietary fibre	g/1000 kcal	6·6	5·7–7·7	9·2	8·1–10·4	< 0·001	7·7	6·5–8·9	8·1	6·7–9·7	< 0·001	7·9	6·5–9·5	7·9	6·6–9·2	0·79
Vitamin A	μgRAE/1000 kcal	366	269–516	530	434–671	< 0·001	427	319–558	497	371–646	< 0·001	446	318–591	469	359–623	< 0·01
Vitamin D	μg/1000 kcal	7·1	4·5–10·6	10·4	6·7–14·7	< 0·001	7·6	4·9–11·7	9·7	6·1–13·9	< 0·001	10·1	6·1–14·4	7·5	5·0–10·9	< 0·001
Vitamin E	mg/1000 kcal	4·1	3·5–4·6	5·3	4·8–5·9	< 0·001	4·5	3·7–5·1	5·0	4·2–5·7	< 0·001	4·5	3·6–5·2	4·9	4·3–5·6	< 0·001
Niacin	mgNE/1000 kcal	9	7–10	11	9–13	< 0·001	9	7–10	11	9–13	< 0·001	9	8–12	10	8–11	0·02
Vitamin B_6_	mg/1000 kcal	0·7	0·6–0·8	0·9	0·8–1·0	< 0·001	0·7	0·6–0·9	0·8	0·7–1·0	< 0·001	0·8	0·7–1·0	0·8	0·7–0·9	0·001
Vitamin B_12_	μg/1000 kcal	4·9	3·5–6·8	6·7	4·7–8·9	< 0·001	5·1	3·7–7·0	6·5	4·4–8·5	< 0·001	6·4	4·4–8·7	5·0	3·6–7·0	< 0·001
Folate	μg/1000 kcal	196	164–223	272	238–313	< 0·001	219	179–258	249	201–299	< 0·001	232	185–282	231	189–276	0·98
Pantothenic acid	mg/1000 kcal	3·7	3·3–4·1	4·5	4·0–4·9	< 0·001	3·9	3·4–4·3	4·3	3·7–4·9	< 0·001	4·1	3·6–4·7	4·1	3·6–4·5	0·09
Vitamin C	mg/1000 kcal	69	53–85	103	86–122	< 0·001	84	66–105	87	66–110	0·31	82	61–103	89	71–111	< 0·001
Na	mg/1000 kcal	2363	2036–2658	2568	2266–2917	< 0·001	2279	1992–2529	2676	2368–3022	< 0·001	2556	2203–2903	2383	1445–1900	< 0·001
K	mg/1000 kcal	1422	1235–1574	1887	1698–2125	< 0·001	1573	1344–1786	1711	1493–2032	< 0·001	1609	1364–1921	1651	1445–1900	0·16
Ca	mg/1000 kcal	319	268–383	415	351–490	< 0·001	347	295–413	384	314–465	< 0·001	366	297–451	362	308–435	0·61
Fe	mg/1000 kcal	4·5	3·9–4·9	5·7	5·1–6·4	< 0·001	4·7	4·1–5·4	5·4	4·7–6·3	< 0·001	5·2	4·5–6·1	4·9	4·3–5·7	< 0·001
Cu	mg/1000 kcal	0·6	0·6–0·7	0·7	0·7–0·8	< 0·001	0·7	0·6–0·7	0·7	0·6–0·8	< 0·001	0·7	0·6–0·8	0·6	0·6–0·7	< 0·001

DP, dietary pattern.

The *P*-values were tested for the low- and high-trend groups.

**Table 4:** Association between dietary patterns and physical performance

DP1, various foods	Model 1			Model 2		
	B	95 % CI	*P*-value	B	95 % CI	*P*-value
Hand grip strength (kg) (*n* 892)	0·02	−0·26, 0·30	0·89	−0·05	−0·33, 0·24	0·76
Timed up-and-go test (sec) (*n* 892)	0·02	−0·17, 0·21	0·85	0·10	−0·09, 0·29	0·29
Usual walking speed (m/sec) (*n* 895)	0·001	−0·012, 0·014	0·84	−0·006	−0·019, 0·006	0·33

DP2, red meats and coffee	Model 1			Model 2		
	B	95 % CI	*P*-value	B	95 % CI	*P*-value
Hand grip strength (kg) (*n* 892)	−0·28	−0·56, –0·002	< 0·05	−0·29	−0·56, –0·01	0·04
Timed up-and-go test (sec) (*n* 892)	0·02	−0·17, 0·21	0·85	0·02	−0·16, 0·19	0·87
Usual walking speed (m/sec) (*n* 895)	< –0·001	−0·013, 0·013	0·97	< 0·001	−0·012, 0·012	0·98

DP3, bread and processed meats	Model 1			Model 2		
	B	95 % CI	*P*-value	B	95 % CI	*P*-value
Hand grip strength (kg) (*n* 892)	−0·09	−0·37, 0·19	0·52	−0·09	−0·37, 0·19	0·54
Timed up-and-go test (sec) (*n* 892)	0·06	−0·13, 0·25	0·53	0·04	−0·14, 0·22	0·64
Usual walking speed (m/sec) (*n* 895)	0·005	−0·008, 0·018	0·47	0·006	−0·007, 0·018	0·38

B, partial regression coefficient; DP, dietary pattern.

Model 1 was adjusted for sex, age, BMI and MMSE.

Model 2 was adjusted for ADL, years of education, economic status, smoking habits, physical activity level, living conditions, medical history and need for long-term care in addition to the variables in Model 1.

## Discussion

This study identified three DP and examined their association with PP (HGS, TUG test and walking speed) in adults aged ≥ 85 years. After adjusting for various confounders, a DP characterised by red meats and coffee consumption (DP2) was negatively associated with HGS. To the best of our knowledge, this is the first study in Asia to examine the relationship between DP and PP in this age group.

The first DP, ‘various foods’, which had the highest contribution in this study, was characterised by the intake of plant foods. This was similar to the ‘healthy’ DP in previous studies, but there was no significant association with PP^(17,18)^. A cohort study of adults aged ≥ 85 years in the UK found that DP (high red meat), characterised by red meat and potatoes, was negatively associated with HGS, similar to the results of this study^(10)^. Although a dramatic westernisation of dietary habits has been pointed out in Japan, the most frequent DP in the 85 years and older group are likely to be similar to the DP of residents in the same country^(17,25)^. However, the similarity of the second DP to DP of the same age group in the UK indicates that changes in dietary habits may occur in this age group^(20)^. It is scientifically interesting that the association with HGS is consistent with evidence from the same age group, as well, although careful observation of the dietary habits of this age group over a longer period is necessary.

HGS is often used as a measure of muscle strength, and the relationship between DP and HGS is considered complex but may be explained by protein intake and intake of nutrients with anti-inflammatory and antioxidant functions^(10)^. Protein is an essential muscle-building nutrient. It has been reported that older adults require more protein for muscle protein synthesis than younger adults; thus, it is clear that adequate protein intake is important for older adults^(26,27)^. The total amount of protein can be broadly divided into plant and animal sources. In terms of lean body mass, animal protein has been suggested to be more beneficial in younger age groups (< 50 years) but has little or no advantage in the middle-aged and older age groups (> 50 years)^(28)^. For the acid–base balance of the diet, it has been suggested that the consumption of high-protein-rich foods can lead to acidosis. Acidosis has been shown to affect protein metabolism, leading to decreased muscle mass by reducing protein synthesis and accelerating muscle protein degradation^(29,30)^. A study in Japan indicated that a higher dietary inflammation index was associated with an increased risk of sarcopenia and with a lower intake of nutrients that protect the muscle from inflammation, but not with over-consumption of nutrients that induce an inflammatory response^(31)^. While the effects of oxidative stress and chronic inflammation of muscle proteins have been discussed, it has also been suggested that micronutrients, with their antioxidant properties, may play a protective role. Vitamin C is particularly known to improve PP^(32)^. It has also been suggested that several micronutrients (such as Fe, Mg and Ca) are associated with muscle protein synthesis, frailty and sarcopenia in the older population^(33,34)^. The critical difference between DP1 and DP2 was the percentage and source of protein intake; DP2 consumed more animal protein than DP1. In addition, for DP1, the source of protein was seafood, such as fish, while for DP2, the main sources were red meat and processed meat. Despite a higher percentage of animal protein intake in DP2, vitamin C intake did not significantly differ between the two groups of DP2. It is also clear that many other micronutrients were less variable than in DP1. This situation may promote chronic inflammation and oxidation of muscles, which may lead to a phenotype of low HGS, promoting a decrease in muscle protein, even if protein intake is sufficient for muscle synthesis. The TUG test and gait speed were not significantly associated with DP. They are measures of comprehensive leg muscle strength. In contrast to HGS, they require not only a single but multiple muscle activities and spatial awareness ability. Therefore, there are complex factors other than nutrition. In addition to kinematic factors such as agility and balance, neurosensory functions such as intelligence and sensation may also be influential^(35,36)^.

This study has several strengths. First, most studies focused on a single food or nutrient, whereas the present study evaluated DP more closely related to real-life dietary habits. Second, this study considered many important confounders, including demographic variables, socio-economic status and lifestyle factors. Third, this is the first study in Asia to examine the relationship between DP and PP in a large population of older adults aged ≥ 85 years, which is very rare worldwide, and age-related confounding may be less than in other studies because the participants were only aged 85 to 89 years. However, this study had several limitations. First, a cross-sectional design was used to examine the relationship between DP and HGS; a causal relationship cannot be inferred. In other words, this study’s results may indicate a trend towards DP characterised by animal foods, such as red meat, in populations with lower HGS. Second, although the BDHQ used in the dietary survey has been validated in older adults of this age group, it may not accurately indicate the dietary habits of this age group because it is not a specialised survey. Finally, since all participants were residents of Kawasaki city, these results may not be representative of other regions. In addition, although the proportion of women should be higher considering the life expectancy in Japan, the ratio of men and women, which was 50 % each, does not rule out the possibility that this age group is not representative of the population. Furthermore, since the sample was limited to older adults requiring up to long-term care level 1, it is unclear whether it represents this age group. Therefore, prospective studies that consider the region, study design and dietary assessment methods are needed to clarify the association shown in the present study.

## Conclusions

A negative relationship was observed between DP characterised by red meat and coffee with HGS in a population aged ≥ 85 years in Japan. It is necessary to examine the association between DP and HGS longitudinally, which showed a negative trend in the present study.
